# A Systematic Analysis of the Clinical Outcome Associated with Multiple Reclassified Desmosomal Gene Variants in Arrhythmogenic Right Ventricular Cardiomyopathy Patients

**DOI:** 10.1007/s12265-023-10403-8

**Published:** 2023-07-07

**Authors:** Emilia Nagyova, Edgar T. Hoorntje, Wouter P. te Rijdt, Laurens P. Bosman, Petros Syrris, Alexandros Protonotarios, Perry M. Elliott, Adalena Tsatsopoulou, Luisa Mestroni, Matthew R. G. Taylor, Gianfranco Sinagra, Marco Merlo, Yuko Wada, Minoru Horie, Jens Mogensen, Alex H. Christensen, Brenda Gerull, Lei Song, Yan Yao, Siyang Fan, Ardan M. Saguner, Firat Duru, Juha W. Koskenvuo, Tania Cruz Marino, Crystal Tichnell, Daniel P. Judge, Dennis Dooijes, Ronald H. Lekanne Deprez, Cristina Basso, Kalliopi Pilichou, Barbara Bauce, Arthur A. M. Wilde, Philippe Charron, Véronique Fressart, Jeroen F. van der Heijden, Maarten P. van den Berg, Folkert W. Asselbergs, Cynthia A. James, Jan D. H. Jongbloed, Magdalena Harakalova, J. Peter van Tintelen

**Affiliations:** 1 Department of Cardiology, Division Heart & Lungs, University Medical Center Utrecht, University of Utrecht, Heidelberglaan 100, 3584 CX Utrecht, The Netherlands; 2 Department of Molecular Biology, Faculty of Natural Sciences, Comenius University in Bratislava, Bratislava, Slovakia; 3 Department of Genetics, University Medical Center Groningen, University of Groningen, Groningen, The Netherlands; 4 Netherlands Heart Institute, Utrecht, The Netherlands; 5 Center for Heart Muscle Disease, Institute of Cardiovascular Science, University College London, London, UK; 6 Nikos Protonotarios Medical Center, 84300 Naxos, Greece; 7 Cardiovascular Institute, University of Colorado Anschutz Medical Campus, Aurora, CO USA; 8 Cardiothoracovascular Department, Azienda Sanitaria-Universitaria Giuliano Isontina (ASUGI), Trieste, Italy; 9 Department of Medicine, Vanderbilt University Medical Center, Nashville, TN USA; 10 Department of Cardiovascular Medicine, Shiga University of Medical Science, Otsu, Japan; 11 Department of Cardiology, Aalborg University Hospital, Aalborg, Denmark; 12 Department of Cardiology, Herlev-Gentofte and Rigshospitalet, Copenhagen University Hospital, Copenhagen, Denmark; 13 Department of Cardiac Sciences, Libin Cardiovascular Institute of Alberta, Calgary, AB Canada; 14 Comprehensive Heart Failure Center (CHFC) and Department of Internal Medicine I, University Hospital Würzburg, Würzburg, Germany; 15 Arrhythmia Center and Clinical EP Laboratory, State Key Laboratory of Cardiovascular Diseases, National Center for Cardiovascular Disease, Fuwai Hospital, Peking Union Medical College-Chinese Academy of Medical Sciences, Beijing, China; 16 Department of Cardiology, University Heart Center, Zurich, Switzerland; 17 Blueprint Genetics, Helsinki, Finland; 18 Department of Medical Biology, CIUSSS Saguenay Lac-St-Jean, Chicoutimi, QC Canada; 19 Division of Cardiology, Department of Medicine, Johns Hopkins University School of Medicine, Baltimore, USA; 20 Division of Cardiology, Department of Medicine, Medical University of South Carolina, Charleston, USA; 21 Department of Genetics, University Medical Center Utrecht, University of Utrecht, Utrecht, the Netherlands; 22 http://guardheart.ernnet.eu; 23 Department of Human Genetics, Amsterdam University Medical Center, University of Amsterdam, Amsterdam, the Netherlands; 24 Department of Cardiac-Thoracic-Vascular Sciences and Public Health, University of Padua, Padua, Italy; 25 Heart Center, Department of Clinical and Experimental Cardiology, Amsterdam University Medical Center, University of Amsterdam, Amsterdam, the Netherlands; 26 APHP, Referral Center for Cardiac Hereditary Diseases, Sorbonne University, Pitié-Salpêtrière Hospital, Paris, France; 27 Department of Cardiology, University of Groningen, University Medical Center Groningen, Groningen, the Netherlands; 28 Institute of Cardiovascular Science, Faculty of Population Health Science, University College London, London, UK; 29 Health Data Research UK and Institute of Health Informatics, University College London, London, UK; 30 Regenerative Medicine Utrecht (RMU), University Medical Center Utrecht, University of Utrecht, Utrecht, The Netherlands; 31 Department of Genetics, University Medical Center Utrecht, University of Utrecht, Utrecht, the Netherlands

**Keywords:** ARVC, Multiple variants, Desmosomal genes, Composite endpoint, Arrhythmia, Genetics

## Abstract

The presence of multiple pathogenic variants in desmosomal genes (*DSC2*, *DSG2*, *DSP*, *JUP*, and *PKP2*) in patients with arrhythmogenic right ventricular cardiomyopathy (ARVC) has been linked to a severe phenotype. However, the pathogenicity of variants is reclassified frequently, which may result in a changed clinical risk prediction. Here, we present the collection, reclassification, and clinical outcome correlation for the largest series of ARVC patients carrying multiple desmosomal pathogenic variants to date (*n* = 331). After reclassification, only 29% of patients remained carriers of two (likely) pathogenic variants. They reached the composite endpoint (ventricular arrhythmias, heart failure, and death) significantly earlier than patients with one or no remaining reclassified variant (hazard ratios of 1.9 and 1.8, respectively). Periodic reclassification of variants contributes to more accurate risk stratification and subsequent clinical management strategy.

**Supplementary Information:**

The online version contains supplementary material available at 10.1007/s12265-023-10403-8.

**Figure:**
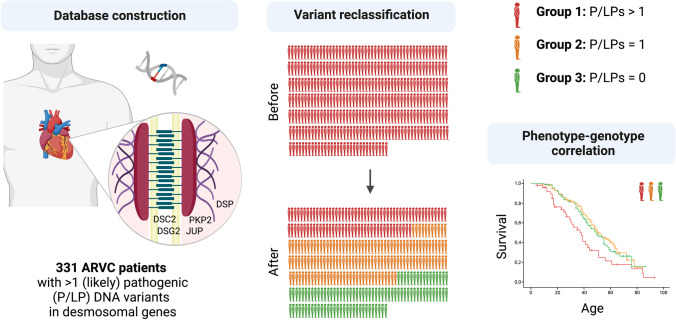
Graphical Abstract

## Introduction

Arrhythmogenic right ventricular cardiomyopathy (ARVC), the main subform of arrhythmogenic cardiomyopathy, is a rare inherited heart disease typically manifesting with ventricular arrhythmias (VA) and gradual fibro-fatty replacement of cardiomyocytes, predominantly in the right ventricle [1, 2]. The phenotype of ARVC is highly variable, and clinical diagnosis requires fulfilling a combination of widely accepted task force criteria related to ventricular structure and function abnormalities, tissue characterisation, repolarisation, depolarisation/conduction, arrhythmias, and family history, including the results of DNA testing [3]. Nearly 50% of ARVC patients are carriers of a pathogenic/likely pathogenic (P/LP) variant in genes encoding desmosomal proteins mainly responsible for cell binding, including desmocollin-2 (*DSC2*), desmoglein-2 (*DSG2*), desmoplakin (*DSP*), junction plakoglobin (*JUP*), and plakophilin-2 (*PKP2*). P/LP variants in non-desmosomal genes are rare and include genes such as desmin (*DES*), phospholamban (*PLN*), and transmembrane protein 43 (*TMEM43*) [4–7].

The phenotypic variability of ARVC is high and still poorly understood, even amongst carriers of an identical P/LP variant. It is assumed that both environmental factors and different genetic backgrounds are involved [1, 8]. Participation in competitive or endurance sports is associated with a worse ARVC prognosis, including earlier presentation of symptoms, worsening of structural abnormalities, higher likelihood of heart failure, and a greater risk of arrhythmias [8]. Worse prognosis, including a higher occurrence and earlier onset of malignant VA, sudden cardiac death, and increased risk of developing left ventricular (LV) dysfunction or heart failure, has also been observed in individuals with more than one P/LP variant [9]. This data suggest a cumulative effect of carrying more than one P/LP variant in the desmosomal genes in ARVC [10].

Previous studies indicate that 2–25% of patients harbour more than one P/LP variant in a desmosomal gene [10–13]. However, since the publication of these studies, variant adjudication criteria have become more strict and large databases like the Genome Aggregation Database (gnomAD) showed that variants previously associated with ARVC occur at a much higher frequency in the population than what would be expected based on disease prevalence. Several studies have been published with additional evidence for or against the pathogenicity of specific variants [14]. Therefore, the true effect of multiple P/LP variants on clinical outcomes remains to be established. After thorough reclassification, we aimed to relate the presence of updated multiple P/LP desmosomal gene variants with clinical outcomes in patients. We hypothesised that after reclassification, patients still having multiple P/LP variants in the five major desmosomal genes have a poorer outcome and prognosis than those with a single P/LP variant or with no P/LP. Therefore, we (a) collected data from published and unpublished patients with more than one desmosomal gene variant underlying ARVC, (b) reclassified these variants, and (c) updated clinical follow-up data to determine the outcome. We aim to contribute to more accurate risk stratification in ARVC patients with more than one P/LP variant.

## Methods

### Inclusion/Exclusion Criteria of the Systematic Literature and Database Search

We searched PubMed (https://www.ncbi.nlm.nih.gov/pubmed/; 2018) and the ARVC Genetic Variants Database (http://www.arvcdatabase.info/) [15] to gather publications containing genetic and clinical information on patients carrying two or more desmosomal gene variants associated with ARVC, including patients having both compound and/or digenic heterozygous variants, as well as at least one homozygous variant. The PubMed search consisted of three combinations of keywords (Supplementary Table 1). We searched for selected terms in the titles and abstracts of the publications. In the ARVC Genetic Variants Database [15], we selected variants co-occurring with other desmosomal variants and collected the respective publications. The search was restricted to English-language literature. To enrich relevant publications, we screened the full text of the pre-selected publications and collected data from publications in which carriers with more than one variant were mentioned. Furthermore, we checked the literature references in these selected manuscripts for carriers of more than one variant. While the term “variant carrier” historically refers to an individual who carries a heterozygous genetic variant without showing any symptoms of the associated recessive condition, here we use it for ARVC patients who carry a variant that can also be associated with an autosomal dominant inheritance.

### ARVC Multiple Variant Database Compilation

**Fig. 1:**
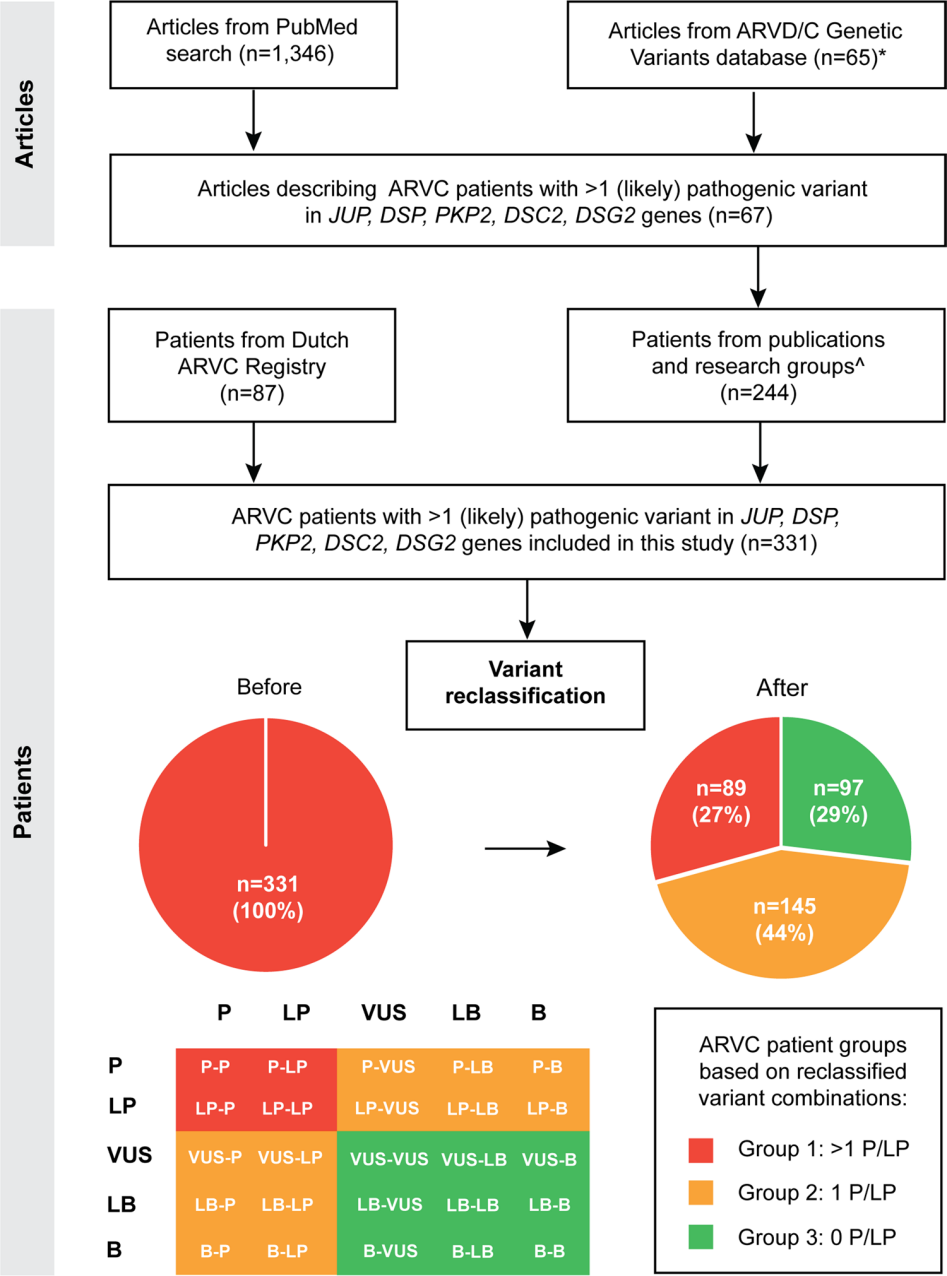
Study workflow and scheme of publication selection with information on patients with two or more ARVC-related desmosomal variants and subsequent patient selection for the database. *61 out of the 65 articles overlapped with the systematic PubMed search, ^number of patients after including information from contacted authors. Patient groups are based on the number of (likely) pathogenic ARVC variants after reclassification; P, pathogenic variant; LP, likely pathogenic variant; VUS, variant with uncertain significance; LB, likely Benign variant; B, benign variant

Using the selected publications (*n* = 67, Fig. 1A), a database of patients with multiple ARVC variants was created. Moreover, unpublished patients with multiple ARVC variants were added, including those from the Dutch ARVC registry (*n* = 87) [16]. Only patients carrying multiple variants (in the same gene or different genes) in *DSC2*, *DSG2*, *DSP*, *JUP*, and *PKP2* genes were included. A predefined extraction sheet was used for data gathering, which included the PubMed identification number of the publication, the number and names of genes tested, and the name of the first author. Whenever available, the following information was extracted from each study: the gene(s) involved, the type of variant, its original classification, cDNA position and change, amino acid position and change, the composition of multiple variants (digenic/compound heterozygous or homozygous), and subject ID. In case genomic coordinates were missing, the TransVar online tool was used to add this information [17]. The following clinical information was collected: sex, the family status of a subject (proband or family member), the age at presentation or diagnosis of disease, the age of the first occurrence of the primary composite endpoint, which consisted of death of any cause, sudden cardiac death, death due to end-stage heart failure, heart transplant and/or left ventricular assist device (LVAD), sustained ventricular tachycardia, ventricular fibrillation, out of hospital cardiac arrest (OHCA), appropriate ICD therapy, and appropriate anti-tachycardia pacing (ATP). If data were missing in publications with five or more carriers of multiple variants, we asked the corresponding authors for follow-up data after the initial publication (Supplementary Table 2). In addition to providing the missing and follow-up data, we asked the authors of the publications with five or more carriers of multiple variants to verify the data collected and to provide data of newly identified yet still unpublished multiple variant carriers when available. The study conforms to the Declaration of Helsinki and was approved by local ethics and/or institutional review boards, and informed consent has been obtained from subjects.

### Variant Reclassification

The variants in patients carrying ostensible multiple P/LP variants were reclassified as pathogenic (P; class 5), likely pathogenic (LP; class 4), uncertain clinical significance (VUS; class 3), likely benign (LB; class 2), or benign (B; class 1) [18]. We used the annotation and visualisation software, Alissa Interpret (Agilent, Santa Clara, CA) and Alamut (Interactive Biosoftware, Rouen, France), to reclassify the variants according to the American College of Medical Genetics (ACMG) and Genomics and the Association for Molecular Pathology criteria [18]. The following summarised classification tree was used. Each variant was first checked if there was already information available in Alissa Interpret Managed Variant List (Agilent, Santa Clara, CA) and Alamut (Interactive Biosoftware, Rouen France), and other publicly available databases (e.g. ClinVar, HGMD), and if so what kind of data. Secondly, consensus rules were followed with respect to truncated (loss-of-function) variants in desmosomal genes. All truncating variants in *DSC2* and *DSG2* and heterozygous truncating variants in *JUP* were assigned VUS if no other supportive information was available. All truncating variants in *DSP* and *PKP2* and recessively inherited truncating variants in *JUP* were considered LP if no other supportive information was available. The last step consisted of the reassessment based on the ACMG criteria [18], thus including in silico prediction data, population data, and, when available: segregation/clinical data, functional data, and gene-specific information including variant type and location.

### Correlation of Severity of the Disease and Type of Multiple Variants

We hypothesised that after reclassification of variants, patients with multiple P/LP variants in the five major desmosomal genes would have poorer outcomes and prognoses than those with a single or no causal P/LP variant remaining. To confirm or reject our hypothesis, we performed a Kaplan–Meier survival analysis based on genotype status. Three groups were made based on the pathogenicity of reclassified ARVC variants (Fig. 1B): patients with two P/LP variants (Group 1), patients with one P/LP variant and a variant with another classification (VUS/LB/B, Group 2), and patients without P/LP variants but with a combination of two VUS/LB/B variants (Group 3). If a patient had three or more ARVC variants, the two variants with the highest pathogenicity class were selected. Homozygous carriers of the Hutterite (*DSC2* c.1660C > T) [19] or Naxos (*JUP* c.2040_2041delGT) [20] founder variants were excluded from Group 1 and were regarded as separate groups. We performed the analysis using Microsoft Excel 2007 (Microsoft, Redmond, WA, USA) and IBM SPSS Statistics 25 (IBM Analytics, Armonk, New York, USA). To compare the outcomes, defined as the age of the first occurrence of one of the events listed in the composite endpoint, in different groups, we created Kaplan–Meier graphs and performed log-rank pairwise comparisons to determine different event-free survival distributions. Variables were included in a multivariable Cox regression model, and correction for proband status and sex was performed.

## Results

### Systematic Literature and Database Search

The systematic PubMed literature search yielded 1 346 publications; the search performed in the ARVD/C Genetic Variants Database yielded 65 articles, of which 61 overlapped with the PubMed literature search. From a total of 1 350 articles, we identified 67 studies (Supplementary Table 2) describing patients with more than one variant believed to be associated with ARVC, which we used to create our study database (Fig. 1A).

### ARVC Multiple Variants Database

Retrieving cases from the selected 67 publications and adding additional cases after contacting the related research groups, including those from the Dutch ARVC registry, resulted in a database containing 500 patients of interest. After de-duplicating patients published in more than one publication, 331 different multiple ARVC variant carriers (135 women and 196 men) were included. Most patients (*n* = 134; 40.5%) were carriers of two heterozygous variants in two different genes (digenic form), 29.3% (*n* = 97) were carriers of a homozygous variant (including 33 homozygous for the Naxos variant and 12 for the Hutterite variant), 24.8% (*n* = 82) were compound heterozygotes with two different variants in one gene, 4.8% (*n* = 16) had a combination of digenic form and compound or homozygous form, while 0.6% (*n* = 2) were carriers of trigenic ARVC variant combinations, i.e. a variant in three different genes (Supplementary Table 3). Before reclassification and excluding the Naxos and Hutterite founder variants, the most frequent combinations of gene variants were *PKP2/DSP* (*n* = 38), *PKP2/DSG2* (*n* = 38), *DSG2/DSG2* (*n* = 33), and *PKP2/PKP2* (*n* = 31, Supplementary Table 4).

After reclassification, 29% of patients (*n* = 96, Supplementary Table 3) were confirmed to have at least two P/LP variants. Of these 96 patients, 33 were homozygous carriers of the Naxos founder variant, 12 were homozygous carriers of the Hutterite variant, and the other 51 carriers of at least two P/LP desmosomal variants were classified as Group 1. Most patients (44%; *n* = 144) had one P/LP variant in combination with a VUS/LB/B variant (Group 2), while 91 patients (28%) had no P/LP variant identified (Group 3, Fig. 1B). After reclassification, the most frequent combination was *PKP2/DSC2* (*n* = 11) followed by *PKP2/DSG2* (*n* = 8); Supplementary Table 4). For an overview of the reclassification of the desmosomal genetic variants, see Supplementary Table 5.

### Correlation Between Severity of the Disease and Type of Multiple Variants

**Fig. 2:**
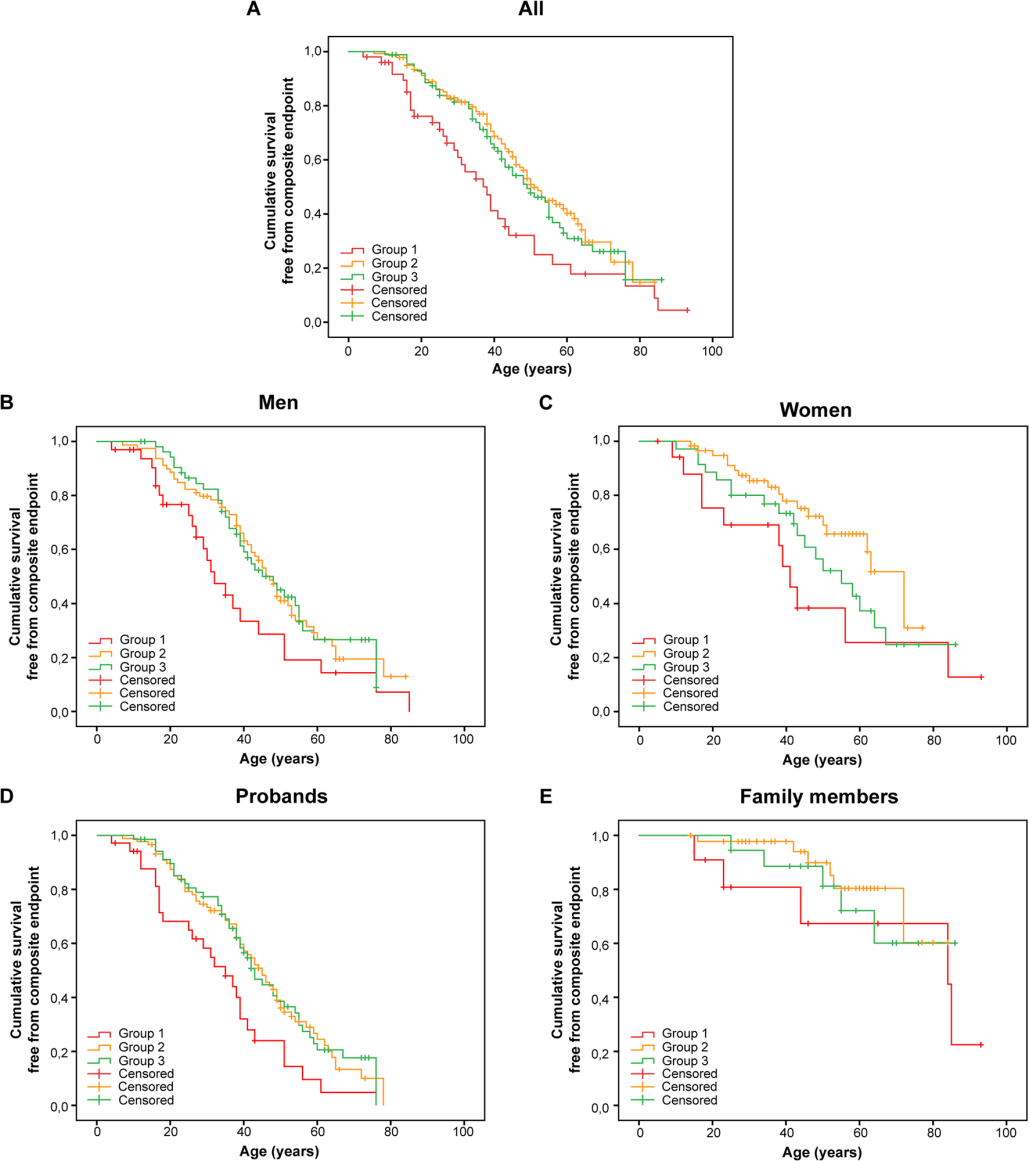
Kaplan–Meier survival analysis. Group 1 (double P/LP variant carriers), Group 2 (one P/LP variant carrier), and Group 3 (no P/LP variant carrier). **A** All. **B, C** All, stratified for sex. **D, E** All, stratified for proband status

The median event-free survival age for Group 1 (double P/LP variants carriers) was 38 years (95% CI, 30–46 years). This was significantly lower than that of Groups 2 (one P/LP variant); 51 years (95% CI, 46–56 years) and 3 (no P/LP variant); 49 years (95% CI, 39–59 years) (Fig. 2A, P = 0.004 and *P* = 0.021 respectively). An overview of the Kaplan–Meier curves stratified for sex and proband status per group is provided in Fig. 2B–E. In a multivariable Cox model, after correcting for proband status and male sex, carrying two P/LP variants remained significantly associated with the composite endpoint with a hazard ratio of 1.9 (95% CI, 1.2–2.9) for Group 1 as compared to Group 2 and a hazard ratio of 1.8 (95% CI, 1.1–2.8) for Group 1 as compared to Group 3 (Supplementary Table 6). For a description of the occurrence of each endpoint, see Supplementary Table 7. An overview of the mean age at presentation or diagnosis, proband status, the occurrence of a composite endpoint, and mean age of occurrence of composite endpoint per group and sex is provided in Supplementary Table 8**.**

### Naxos and Hutterite Founder Variants Carriers

**Fig. 3:**
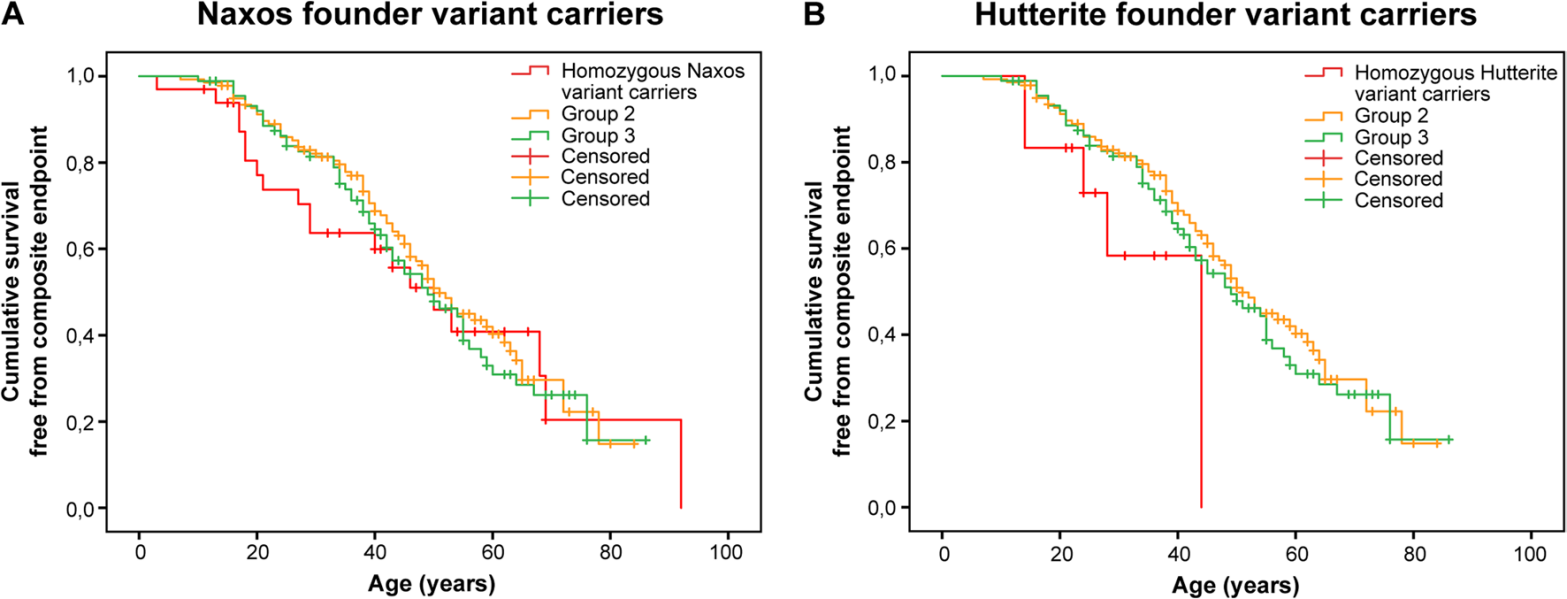
Kaplan–Meier survival analysis of homozygous Naxos and homozygous Hutterite founder variant carriers. **A** Kaplan–Meier curves of homozygous Naxos variant carriers, Group 2 (one P/LP variant carrier) and Group 3 (no P/LP carrier). **B** Kaplan–Meier curves of homozygous Hutterite variant carriers, Group 2 (one or none P/LP variant carrier), and Group 3 (no P/LP variant carrier)

The median event-free survival age for the patient populations homozygous for Naxos or Hutterite founder variants was 50 years (95% CI, 37–63 years) and 44 years (95% CI, NA), respectively, while the median age for patients from Group 2 and 3 was 51 years (95% CI, 46–56 years) and 49 years (95%, CI, 39–59 years, Fig. 3A/B). There were no significant differences in outcome between both homozygous founder variant carriers versus Group 2 or 3. In a multivariable Cox model, after correcting for proband status and male sex, being a homozygous carrier of the Naxos variant was significantly associated with the composite endpoint with a hazard ratio of 1.8 (95% CI, 1.03–2.99) compared to Group 2 (carriers of 1 P/LP), but was not associated compared to Group 3 (no P/LP carriers) with a hazard ratio of 1.6 (95% CI, 0.9–2.9, Supplementary Table 9).

Being a homozygous carrier of the Hutterite variant was significantly associated with the composite outcome in a multivariable Cox model, after correcting for proband status and male sex, compared to Group 2 with a hazard ratio of 6.9 (95% CI, 2.3–20.2) as well as compared to Group 3 with a hazard ratio of 5.5 (95% CI, 1.7–17.4, Supplementary Table 10).

## Discussion

This study aimed to establish a reliable estimate of the effect of multiple reclassified P/LP desmosomal gene variants on the severity of ARVC. Published series often have a limited size, and variant classification rules have evolved and become more stringent over the last years [9, 12, 13]. Therefore, we pooled the results of patients reported to have multiple P/LP variants from a large number of studies in a systematic quantitative analysis. Importantly, we performed the analysis after uniform reclassification of all variants identified and an update of clinical follow-up.

In this series of patients with multiple (reclassified) P/LP variants, the largest such study to our knowledge, we showed that this group reached the composite endpoint, consisting of death of any cause, sudden cardiac death, death due to end-stage heart failure, heart transplant and/or left ventricular assist device (LVAD), sustained ventricular tachycardia, ventricular fibrillation, out of hospital cardiac arrest (OHCA), appropriate ICD-therapy, and appropriate anti-tachycardia pacing (ATP), significantly earlier than those with one or no P/LP variant: at median age 38 years as compared to 51 and 49 years, respectively. Also, after correcting for proband status and male sex, carrying two P/LP variants remained significantly associated with reaching the composite endpoint with hazard ratios of 1.9 and 1.8 as compared to those with one and no P/LP variants, respectively. Putting it simply, the presence of multiple P/LP variants portends earlier and more severe disease.

Previous studies also showed that endpoints were reached earlier in patients with multiple P/LP variants. Bhonsale et al. described 22 patients with multiple pathogenic variants in ARVC-associated genes. They had significantly earlier occurrence of sustained VT/VF, lower VT-/VF-free survival, a fivefold increase in the risk of developing LV dysfunction, and more frequent cardiac transplantation when compared with those with only a single (likely) pathogenic variant [13]. In a study by Rigato et al., in 7 compound and 14 digenic heterozygous patients, compound/digenic heterozygosity was an independent predictor of lifetime arrhythmic events with a hazard ratio of 3.71 (95% CI, 1.548.92; *P* = 0.003) [9]. Moreover, Bauce et al. noted a higher extent of disease phenotype in terms of LV (*P* = 0.025) and RV dilatation (trend toward statistical significance *P* = 0.051) in three index cases and seven family members with multiple (likely) pathogenic variants [12].

Reclassification revealed that only 29% of published cases with ostensibly two or more “mutations” actually had two P/LP variants after reclassification. Reclassifications were based on several observations or their combinations: (1) improved population frequency information as a result of a higher number of control exomes/genomes available (e.g. ExAc database of ~ 60,000 exomes vs. gnomAD database of ~ 140,000 exomes and genomes), (2) the use of improved computational tools, (3) newly available patient information either published or in databases (e.g. HMD), such as the identification of more independent probands with similar phenotypes, (4) the availability of (more) co-segregation data (underscoring pathogenicity), or lack of co-segregation (disputing pathogenicity), or (5) novel functional data supporting pathogenicity. In a recent study, rare variants associated with arrhythmogenic cardiomyopathy were reclassified in 31% of cases after 5 years of follow-up since 1996 [21]. The authors suggest a periodic genetic reanalysis of rare variants every 5 years. A recent study by Costa et al. showed that 59% of 80 ARVC-related variants were reclassified with a presumed clinically relevant reclassification in 33 variants (41%). This led to 10% of patients being downgraded from a definite diagnosis of ARVC to borderline/possible disease [14]. It is recommended to exercise caution, particularly when considering variants that were classified prior to 2015/2017 (ExAC/gnomAD).

This modified classification is relevant not only for patient diagnosis, prognosis, and treatment but also for genetic cascade screening of family members and, potentially, prenatal or pre-implantation diagnostics, even though many aspects of penetrance of desmosomal gene variants are still poorly understood [22]; i.e. what is the penetrance of a single P/LP desmosomal gene variant in a family with ARVC, in an individual identified in a population study, or a heterozygous relative of an index-patient with multiple P/LP variants. The impact and importance of correct classification of genetic variants have recently also been demonstrated in Brugada syndrome patients, where functional characterisation/validation helped correctly classify sodium voltage-gated channel alpha subunit 5 (*SCN5A*) variants. Loss of function (LOF) variants were associated with earlier onset of lethal arrhythmic events and, thus, a worse prognosis as compared to non-LOF *SCN5A* variants [23]. This was further corroborated by Ciconte et al., where proven LP/P *SCN5A* variant carriers had a higher prevalence of aborted cardiac arrest or spontaneous ventricular tachycardia/fibrillation requiring ICD therapy [24].

In our dataset of patients carrying multiple desmosomal variants, we used guidelines based on the current ACMG criteria to identify patients carrying two or more reclassified P/LP variants in the desmosomal genes. This refinement showed that genotype, i.e. carrying multiple desmosomal P/LP variants, has an additional effect on the outcome. The control groups consisted of a group of patients (Group 2) carrying one desmosomal P/LP variant combined with a VUS or B/LB and another group of patients (Group 3) with a combination of two variants classified as either VUS or B/LB. Of note, no difference in outcome was found between these two latter groups. This could be because both groups are still too heterogeneous, and the effect of these variants remains unknown, emphasizing the need for additional functional and co-segregational analyses [14]. Additionally, some VUS could have a high or low suspicion of pathogenicity. Combining this group with B/LB or P/LP variants could affect the outcome. To assess a possible effect, we divided the group that carried a VUS into subgroups where patients carry an additional P/LP variant (*n* = 99) or additional VUS (*n* = 63) or B/LB variant (*n* = 16). If there are any possible suspicious VUS in those groups, the outcome would be expected to be more severe in the group with a P/LP. However, as shown in the Kaplan–Meier survival curves (Supplementary Fig. 1), there were no differences observed. After correcting for sex and proband status, an HR of 1.1 (95% CI, 0.7 and 1.7) for the group carrying a VUS and a P/LP variant compared to the group of carriers with two VUS. An HR of 1.1 (95% CI, 0.4 and 2.6) was found for the group carrying a VUS and B/LB variant compared to the group with two VUS, and when comparing the group carrying a VUS and a P/LP variant to the group carriers with a VUS and a B/LB, the HR was 0.9 (95% CI, 0.4 and 2.2). Therefore, although we cannot exclude that there is an effect of grouping these VUS with B/LB variants or P/LP variants, we did not find an effect of possible highly suspicious VUS in these groups.

We did not include carriers of multiple variants in genes other than the five desmosomal genes. The reason is that ARVC is considered a desmosomal disease, and the majority of the (likely) pathogenic variants are found in the desmosomal genes [25]. A recent international ARVC gene-curation effort reported that, next to *TMEM43*, only the five desmosomal genes had definite evidence for an association with ARVC [6].

Finally, the recessive *DSC2* c.1660C > T (p.Q554*) Hutterite and *JUP* c.2040_2041delGT (p.W680Gfs*11) Naxos founder variants also showed to be associated with a worse outcome after correcting for male sex and proband status, although this was not significant for the homozygous Naxos variant carriers versus Group 3 (no P/LP variants).

### Limitations and strengths

Our analyses are limited by the incompleteness of reported data in the original studies. In addition, not all relevant genes were tested in all studies included. Therefore, the possibility cannot be excluded that a small number of patients included in our study had additional P/LP variants in those unanalysed genes. This may have led to an overestimation of the severity of phenotypes in subjects with one or no P/LP variants. Furthermore, the patients included in this cohort are from several large studies and registries describing ARVC patients and their family members. Unfortunately, we lack individual data regarding definite ARVC diagnosis or which task force criteria are fulfilled. Especially for family members, it is possible that they did not meet the diagnostic criteria or were asymptomatic. However, by analysing probands and family members separately, we believe this is of limited consequence since probands carrying two or more P/LP variants significantly affected the outcome (Fig. 2D). Also, data regarding ethnicity and specific clinical data, like the history of exercise, cardiac function (ejection fraction), or the presence of late gadolinium enhancement on MRI, were generally unavailable. Additionally, segregation data might have become available in unknown centres for us, which could have played a role in the correct classification. Another potential limitation could be that data on the localisation of variants (in *trans* or *cis*) was generally unavailable in patients with compound heterozygous variants.

The ultimate strength of this study is the large sample size (multinational cohort) and that we performed our analysis after uniform and stringent reclassification of all published variants that were formerly qualified as “mutations” to overcome differences in variant classification and to establish the real impact of multiple P/LP variants on the ARVC phenotype. This reclassification eventually led to 29% of patients having two P/LP variants. Usually, around 17% of variants have conflicting interpretations, and their real impact on phenotype is unknown [26]. Also, for ARVC, it is known that several variants that were initially published as “mutations” or likely “mutations” did not turn out to be causative after more extensive functional and population studies (e.g. see [27–29]). It is important to note that our study focused on ARVC patients with multiple variants (“mutations”) prior to variant reclassification, and thus the comparisons were derived from a specific subset of patients. Therefore, there could be intrinsic differences in the outcomes of interest for this particular population as compared to the broader ARVC population with a single P/LP desmosomal variant or no P/LP variants.

## Conclusion

After reclassification of variants identified in 331 patients with two ostensible desmosomal “mutations” from a large multicenter international series, only 29% carried two P/LP variants in desmosomal genes according to current classification criteria. Event-free survival analysis in 51 individuals of this group revealed these patients had a worse outcome: a median event-free survival at the age of 38 years compared to 51 and 49 years for patients with one or no P/LP variant, respectively. Carrying two P/LP variants is significantly associated with reaching the composite endpoint (ventricular arrhythmias, heart failure, or death). These results corroborate previous findings that carrying more than one P/LP variant contributes to the risk of developing life-threatening cardiac events in ARVC patients and contributes to a more accurate risk assessment in ARVC patients.

These findings underscore the clinical relevance and importance of periodically reclassifying all relevant ARVC genes in patients. Knowledge of correctly classified variants in relevant genes is of great importance to more accurately predict the future development of the disease and the identification of high-risk patients, even in the early stages of disease manifestation.

### Supplementary Information

Below is the link to the electronic supplementary material.

Fig. S1 Kaplan–Meier survival analysis on three groups based on the carriership of at least a single VUS and another variant: VUS & VUS, VUS & P/LP, and VUS & B/LB (PNG 71 kb)

High Resolution (TIF 12.5 mb)

Table S1 (XLSX 11 kb)

Table S2 (XLSX 17 kb)

Table S3 (XLSX 12 kb)

Table S4 (XLSX 12 kb)

Table S5 (XLSX 64 kb)

Table S6 (XLSX 11 kb)

Table S7 (XLSX 11 kb)

Table S8 (XLSX 12 kb)

Table S9 (XLSX 12 kb)

Table S10 (XLSX 12 kb)

## Abbreviations

ATP: Appropriate anti-tachycardia pacing
ARVC: Arrhythmogenic right ventricular cardiomyopathy
ACMG/AMP: American College of Medical Genetics and Genomics and Association for Molecular Pathology
B: Benign
DSC2: Desmocollin-2
DSG2: Desmoglein-2
DCM: Dilated cardiomyopathy
DSP: Desmoplakin
ICD: Implantable cardioverter defibrillator
LB: Likely benign
LP: Likely pathogenic
LVAD: Left ventricular assist device
JUP: Plakoglobin
OHCA: Out of hospital cardiac arrest
P: Pathogenic
P/LP: Pathogenic/likely pathogenic
PKP2: Plakophilin-2
SCD: Sudden cardiac death
VA: Ventricular arrhythmias
VUS: Variant of unknown significance

## References

[1] Hoorntje ET, Te Rijdt WP, James CA, Pilichou K, Basso C, Judge DP (2017). Arrhythmogenic cardiomyopathy: pathology, genetics, and concepts in pathogenesis. Cardiovasc Res..

[2] Basso C, Corrado D, Marcus FI, Nava A, Thiene G (2009). Arrhythmogenic right ventricular cardiomyopathy. Lancet..

[3] Marcus FI, McKenna WJ, Sherrill D, Basso C, Bauce B, Bluemke DA (2010). Diagnosis of arrhythmogenic right ventricular cardiomyopathy/dysplasia: proposed modification of the Task Force Criteria. Eur Heart J..

[4] van Tintelen JP, Van Gelder IC, Asimaki A, Suurmeijer AJ, Wiesfeld AC, Jongbloed JD (2009). Severe cardiac phenotype with right ventricular predominance in a large cohort of patients with a single missense mutation in the DES gene. Heart Rhythm.

[5] Merner ND, Hodgkinson KA, Haywood AF, Connors S, French VM, Drenckhahn JD (2008). Arrhythmogenic right ventricular cardiomyopathy type 5 is a fully penetrant, lethal arrhythmic disorder caused by a missense mutation in the TMEM43 gene. Am J Hum Genet..

[6] James CA, Jongbloed JDH, Hershberger RE, Morales A, Judge DP, Syrris P (2021). International evidence based reappraisal of genes associated with arrhythmogenic right ventricular cardiomyopathy using the clinical genome resource framework. Circ Genom Precis Med..

[7] van der Zwaag PA, van Rijsingen IAW, Asimaki A, Jongbloed JDH, van Veldhuisen DJ, Wiesfeld ACP (2012). Phospholamban R14del mutation in patients diagnosed with dilated cardiomyopathy or arrhythmogenic right ventricular cardiomyopathy: evidence supporting the concept of arrhythmogenic cardiomyopathy. Eur J Heart Failure.

[8] James, C. A., Bhonsale, A., Tichnell, C., Murray, B., Russell, S. D., Tandri, H., et al. (2013). Exercise increases age-related penetrance and arrhythmic risk in arrhythmogenic right ventricular dysplasia/cardiomyopathy-associated desmosomal mutation carriers. J Am College Cardiol. 62(14). 10.1016/j.jacc.2013.06.033. 10.1016/j.jacc.2013.06.033 PMC3809992 23871885

[9] Rigato I, Bauce B, Rampazzo A, Zorzi A, Pilichou K, Mazzotti E (2013). Compound and digenic heterozygosity predicts lifetime arrhythmic outcome and sudden cardiac death in desmosomal gene-related arrhythmogenic right ventricular cardiomyopathy. Circ Cardiovasc Genet..

[10] Xu T, Yang Z, Vatta M, Rampazzo A, Beffagna G, Pilichou K (2010). Compound and digenic heterozygosity contributes to arrhythmogenic right ventricular cardiomyopathy. J Am College Cardiol..

[11] Bhonsale A, James CA, Tichnell C, Murray B, Madhavan S, Philips B (2013). Risk stratification in arrhythmogenic right ventricular dysplasia/cardiomyopathy-associated desmosomal mutation carriers. Circ Arrhythmia Electrophysiol..

[12] Bauce B, Nava A, Beffagna G, Basso C, Lorenzon A, Smaniotto G (2010). Multiple mutations in desmosomal proteins encoding genes in arrhythmogenic right ventricular cardiomyopathy/dysplasia. Heart Rhythm..

[13] Bhonsale A, Groeneweg JA, James CA, Dooijes D, Tichnell C, Jongbloed JD (2015). Impact of genotype on clinical course in arrhythmogenic right ventricular dysplasia/cardiomyopathy-associated mutation carriers. Eur Heart J..

[14] Costa S, Medeiros-Domingo A, Gasperetti A, Akdis D, Berger W, James CA (2021). Impact of genetic variant reassessment on the diagnosis of arrhythmogenic right ventricular cardiomyopathy based on the 2010 Task Force Criteria. Circ Genom Precis Med..

[15] Lazzarini E, Jongbloed JD, Pilichou K, Thiene G, Basso C, Bikker H (2015). The ARVD/C genetic variants database: 2014 update. Hum Mutat..

[16] Bosman LP, Verstraelen TE, van Lint FHM, Mgpj C, Groeneweg JA, Mast TP (2019). The Netherlands Arrhythmogenic Cardiomyopathy Registry: design and status update. Netherlands Heart J.

[17] Zhou W, Chen T, Chong Z, Rohrdanz MA, Melott JM, Wakefield C (2015). TransVar: a multilevel variant annotator for precision genomics. Nat Methods..

[18] Richards S, Aziz N, Bale S, Bick D, Das S, Gastier-Foster J (2015). Standards and guidelines for the interpretation of sequence variants: a joint consensus recommendation of the American College of medical Genetics and Genomics and the Association for Molecular Pathology. Genet Med..

[19] Gerull B, Kirchner F, Chong JX, Tagoe J, Chandrasekharan K, Strohm O (2013). Homozygous founder mutation in desmocollin-2 (DSC2) causes arrhythmogenic cardiomyopathy in the Hutterite population. Circ Cardiovasc Genet..

[20] Kırali K. Cardiomyopathies: Types and treatments. BoD—Books on Demand. 2017;470. 10.5772/62816.

[21] Vallverdú-Prats M, Alcalde M, Sarquella-Brugada G, Cesar S, Arbelo E, Fernandez-Falgueras A, … Campuzano O. Rare variants associated with arrhythmogenic cardiomyopathy: reclassification five years later. J Personal Med. 2021;11(3). 10.3390/jpm11030162. 10.3390/jpm11030162 PMC7996798 33652588

[22] Carruth ED, Young W, Beer D, James CA, Calkins H, Jing L (2019). Prevalence and electronic health record-based phenotype of loss-of-function genetic variants in arrhythmogenic right ventricular cardiomyopathy-associated genes. Circ Genom Precis Med..

[23] Ishikawa T, Kimoto H, Mishima H, Yamagata K, Ogata S, Aizawa Y (2021). Functionally validated SCN5A variants allow interpretation of pathogenicity and prediction of lethal events in Brugada syndrome. Eur Heart J..

[24] Ciconte G, Monasky MM, Santinelli V, Micaglio E, Vicedomini G, Anastasia L (2021). Brugada syndrome genetics is associated with phenotype severity. Eur Heart J..

[25] Groeneweg JA, van der Heijden JF, Dooijes D, van Veen TA, van Tintelen JP, Hauer RN (2014). Arrhythmogenic cardiomyopathy: diagnosis, genetic background, and risk management. Netherlands Heart J..

[26] Rehm HL, Berg JS, Brooks LD, Bustamante CD, Evans JP, Landrum MJ (2015). ClinGen–the Clinical Genome Resource. N Engl J Med..

[27] Posch MG, Posch MJ, Perrot A, Dietz R, Ozcelik C (2008). Variations in DSG2: V56M, V158G and V920G are not pathogenic for arrhythmogenic right ventricular dysplasia/cardiomyopathy. Nat Clin Pract Cardiovasc Med..

[28] Christensen AH, Kamstrup PR, Gandjbakhch E, Benn M, Jensen JS, Bundgaard H (2016). Plakophilin-2 c.419C>T and risk of heart failure and arrhythmias in the general population. Eur J Human Genet.

[29] Gandjbakhch E, Charron P, Fressart V, de la Grandmaison GL, Simon F, Gary F (2011). Plakophilin 2A is the dominant isoform in human heart tissue: consequences for the genetic screening of arrhythmogenic right ventricular cardiomyopathy. Heart..

