# Supplementary figures and images for: A Systematic Analysis of the Clinical Outcome Associated with Multiple Reclassified Desmosomal Gene Variants in Arrhythmogenic Right Ventricular Cardiomyopathy Patients
Source: J Cardiovasc Transl Res. 2023 Jul 7;16(6):1276–86. doi: 10.1007/s12265-023-10403-8 (PMC10721666; doi:10.1007/s12265-023-10403-8)

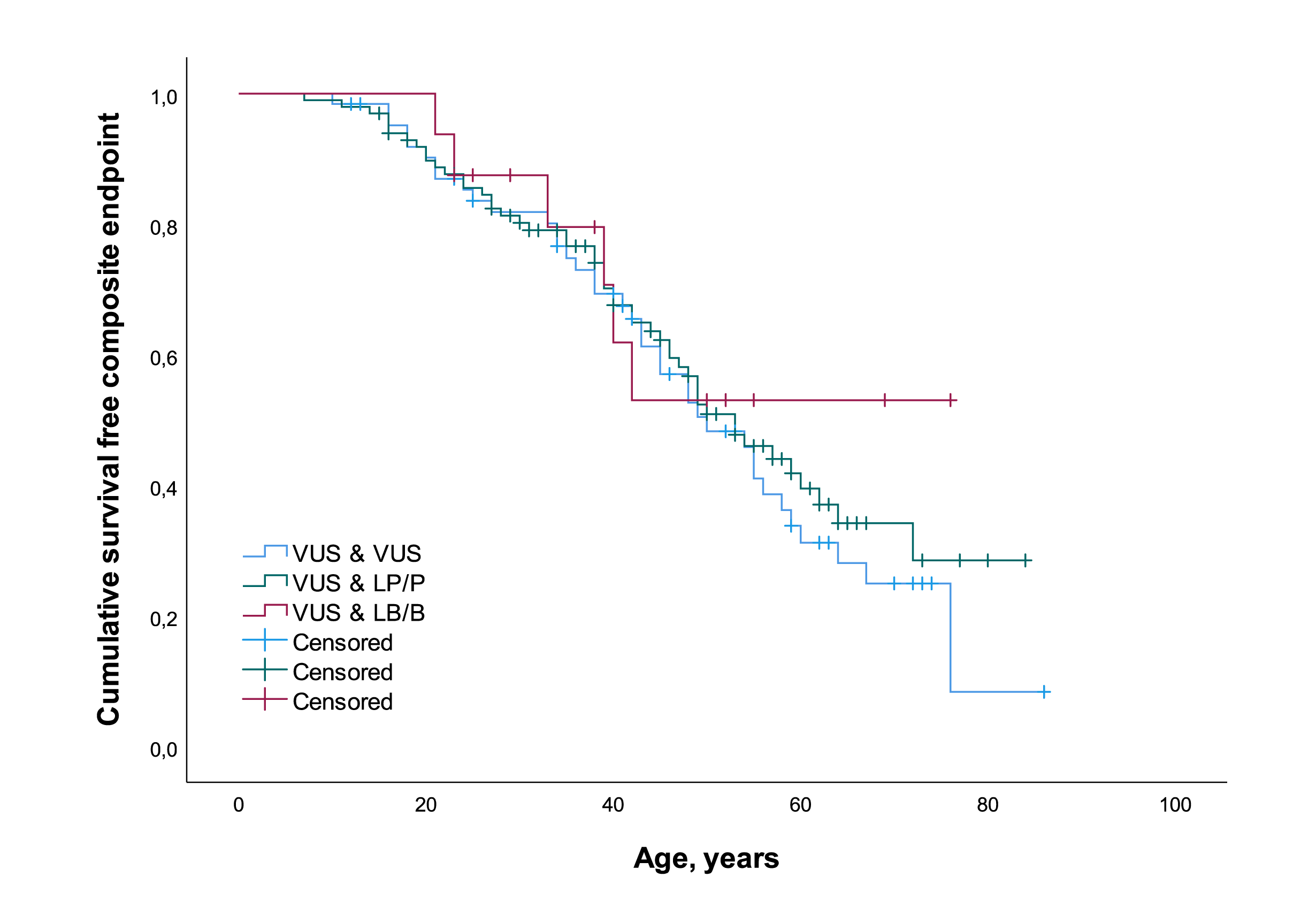

Supplement: Supplementary file 1 — Kaplan–Meier survival analysis on three groups based on the carriership of at least a single VUS and another variant: VUS & VUS, VUS & P/LP, and VUS & B/LB (PNG 71 kb) [file 12265_2023_10403_Fig4_ESM.png]

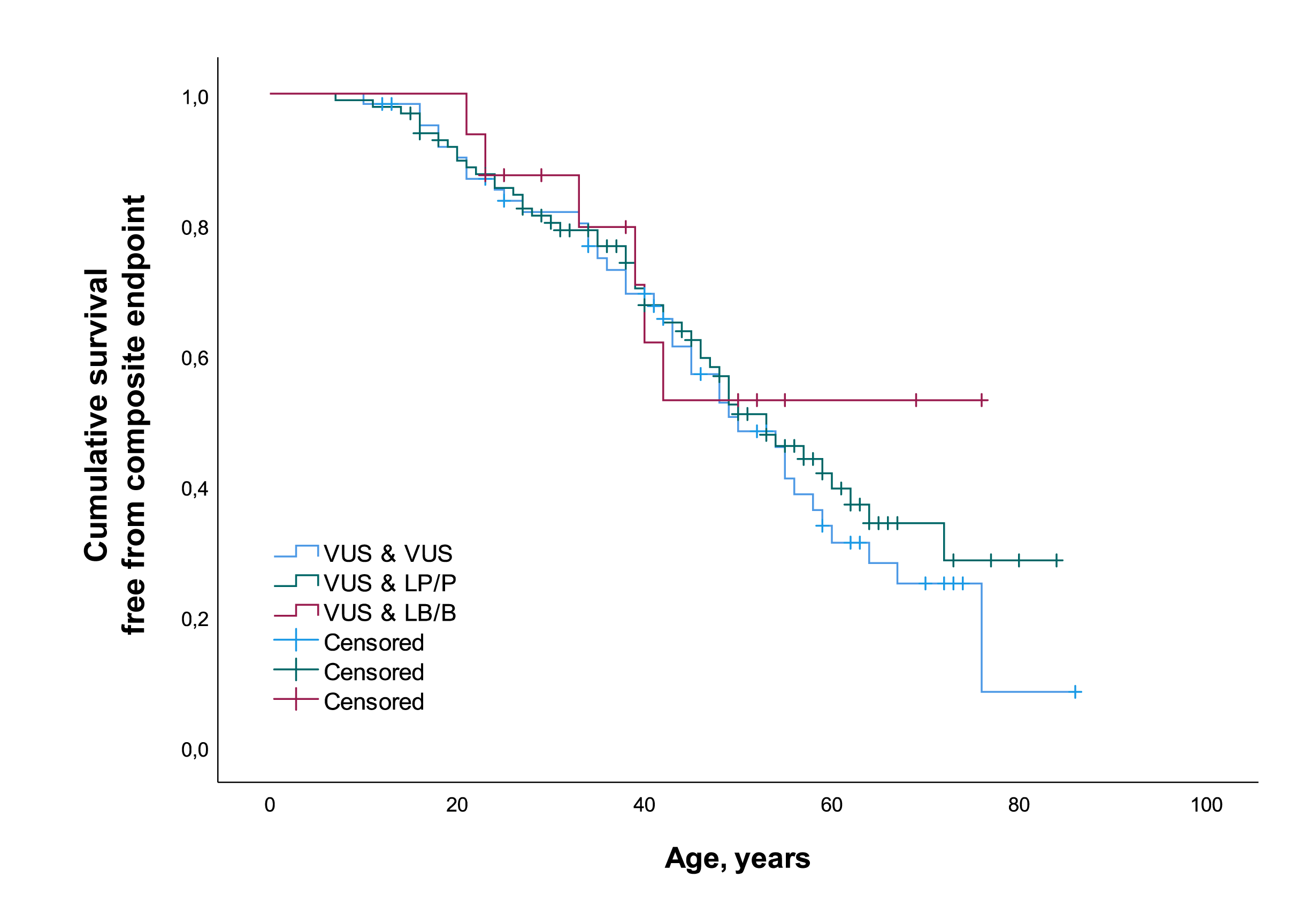

Supplement: Supplementary file 2 — High Resolution (TIF 12.5 mb) [file 12265_2023_10403_MOESM1_ESM.tif]
